# Supplementary material for: Breaking the Coupled Cluster Barrier for Machine-Learned Potentials of Large Molecules: The Case of 15-Atom Acetylacetone
Source: J Phys Chem Lett. 2021 May 19;12(20):4902–9. doi: 10.1021/acs.jpclett.1c01142 (PMC8279733; doi:10.1021/acs.jpclett.1c01142)
Supplement: Supplementary file 1 — jz1c01142_si_001.pdf [file jz1c01142_si_001.pdf]

Supporting Information: Breaking the Coupled Cluster Barrier for Machine Learned Potentials of Large Molecules: The Case of 15-atom Acetylacetone

Chen Qu,[†] Paul L. Houston,[*,‡] Riccardo Conte,[*,¶] Apurba Nandi,[§] and Joel M. Bowman[*,§]

†*Department of Chemistry & Biochemistry, University of Maryland, College Park, Maryland 20742, U.S.A.*

‡*Department of Chemistry and Chemical Biology, Cornell University, Ithaca, New York 14853, U.S.A. and Department of Chemistry and Biochemistry, Georgia Institute of Technology, Atlanta, Georgia 30332, U.S.A*

¶*Dipartimento di Chimica, Università Degli Studi di Milano, via Golgi 19, 20133 Milano, Italy*

§*Department of Chemistry and Cherry L. Emerson Center for Scientific Computation, Emory University, Atlanta, Georgia 30322, U.S.A.*

E-mail: plh2@cornell.edu; riccardo.conte1@unimi.it; jmbowma@emory.edu

Numbering Scheme

The numbering scheme for the difference potential fit is shown in Fig. S1. The permutational symmetry label for this is 1, 2, 5, 7. This indicates that the 7 H atoms, atom numbers 9, 10, 11, 12, 13, 14, 15 are treated as permutable, as are the 2 O atoms, numbers 2 and 3, and the 5 C atoms, 4, 5, 6, 7, 8. The transferring atom H, atom 1, is treated as distinguishable.

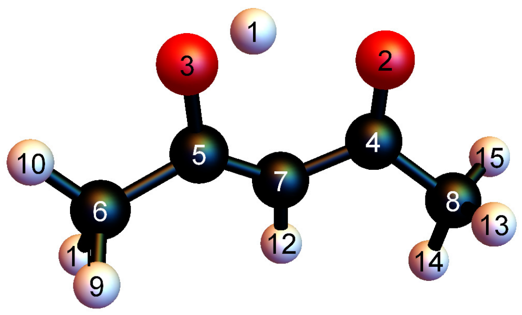

Figure S1: Numbering scheme used in the PES difference fit of symmetry 1,2,5,7.

Benchmark Calculations

In addition to the LCCSD(T)-F12 energies calculated for the PES data base, we performed two benchmark calculations at the global minimum and at the saddle point for H-atom transfer. These two calculations found the optimum geometry and energy and determined as well the harmonic vibrational frequencies and normal coordinates. While the LCCSD(T)-F12 calculations for just the energy at a single geometry took approximately 30 minutes using 12 cores of the 2.4 GHz Intel Xeon processors, the full optimization and frequency calculations took on the order of 73 days using the same number of processors. This computational cost certainly underscores the infeasibility of doing even approximate LCCSD(T) calculations for an AcAc PES.

The harmonic frequencies of the global minimum and saddle point from the MP2 PES (V_{LL}), the corrected PES ($V_{LL\rightarrow CC}$) using 1935 training points, and direct LCCSD(T)-F12

Table S1: Harmonic frequencies (in cm^{-1}) of the global minimum and H-transfer saddle point of acetylacetone from the indicated sources. Training was done with 1935 LCCSD(T) energies.

	Global Minimum			Saddle Point		
mode	LCCSD(T)	$V_{LL\rightarrow CC}$	V_{LL}	LCCSD(T)	$V_{LL\rightarrow CC}$	V_{LL}
1	113	97	97	1278i	1082i	921i
2	133	120	119	100	57	53
3	169	157	153	121	62	57
4	197	191	189	165	159	156
5	236	227	229	198	197	198
6	372	359	364	289	286	285
7	392	389	390	412	416	417
8	505	502	507	537	530	531
9	554	570	567	540	540	539
10	643	652	650	578	589	580
11	654	657	656	661	654	645
12	793	801	803	767	739	740
13	919	881	921	781	751	756
14	942	912	936	949	947	953
15	951	934	942	992	981	979
16	1010	1005	1014	1035	1032	1039
17	1040	1044	1048	1037	1039	1043
18	1050	1052	1058	1054	1059	1060
19	1072	1069	1071	1067	1060	1062
20	1192	1192	1200	1195	1182	1189
21	1276	1270	1290	1308	1250	1223
22	1393	1377	1384	1341	1341	1347
23	1405	1404	1399	1406	1401	1409
24	1424	1433	1433	1413	1418	1422
25	1462	1450	1470	1481	1490	1496
26	1480	1486	1494	1487	1491	1496
27	1483	1489	1497	1488	1494	1500
28	1488	1499	1505	1491	1496	1502
29	1502	1508	1512	1569	1560	1567
30	1670	1662	1655	1613	1617	1629
31	1709	1705	1704	1624	1648	1670
32	3047	3058	2855	1904	1744	1685
33	3052	3080	3095	3054	3058	3098
34	3118	3122	3099	3057	3079	3099
35	3122	3170	3178	3130	3179	3190
36	3157	3178	3187	3132	3180	3190
37	3165	3203	3208	3154	3196	3207
38	3220	3210	3218	3156	3197	3208
39	3257	3248	3258	3241	3273	3282

calculations are listed in Table S1. For most of the modes, the differences between V_{LL} and $V_{LL\rightarrow CC}$ frequencies are small, but for mode 32 of GM (OH stretch) and the imaginary-frequency mode of the H-transfer SP, the improvement of the Δ-ML PES is significant. Again, the 4 Δ-ML PESs based on different training sets achieved similar MAE in frequencies (around 13 cm^{-1} for GM and 25 cm^{-1} for H-transfer SP, see Table 1 of the main text), and that is a significant improvement over the low-level PES, which has MAEs of 17.3 and 35.6 cm^{-1} for GM and H-transfer SP, respectively.

Fitting Precision

Comparison between the geometries of two stationary points from the Δ-ML PES and those optimized at LCCSD(T)-F12 level of theory.

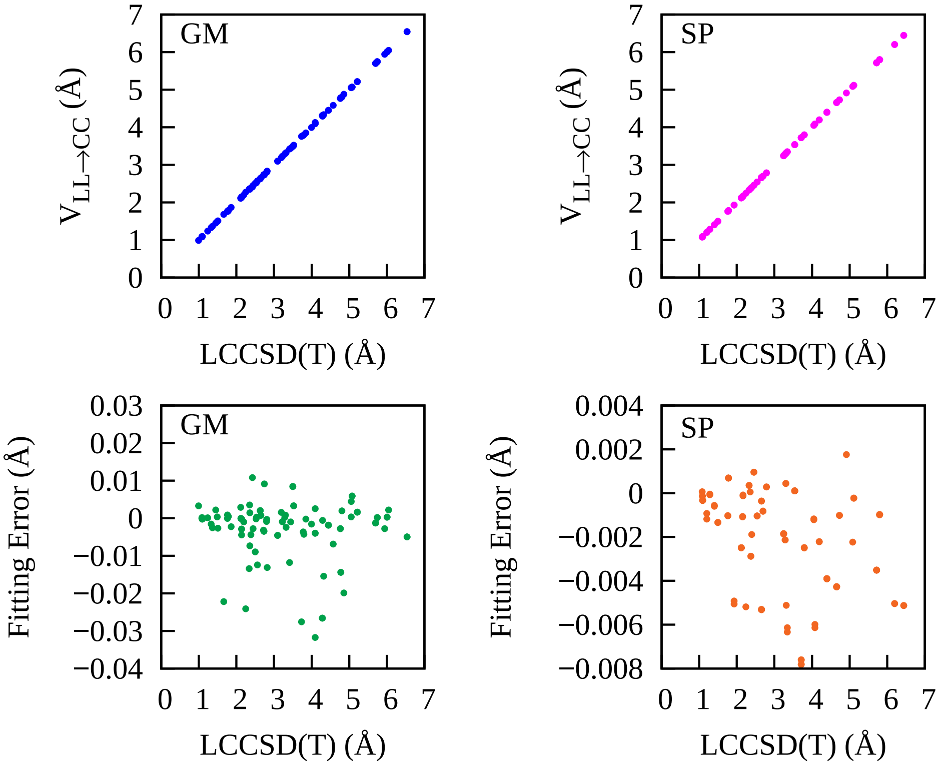

Fig. S2: Plot of fitting errors in inter-nuclear distances. The upper panel represents the plot of Δ-ML PES distance vs direct LCCSD(T) distance and the lower panel represents the fitting error at each distance.
